# Non-inferiority of sleep position therapy compared to positive airway pressure therapy with regard to daytime sleepiness in patients with mild to moderate position-dependent obstructive sleep apnoea (POSA): study protocol for a multicentre randomised cross-over trial

**DOI:** 10.1186/s13063-025-09007-1

**Published:** 2025-08-18

**Authors:** Nina Timmesfeld, Anja Neumann, Frederik Valbert, Jürgen Wasem, Alexandra Spillner, Christoph Schöbel

**Affiliations:** 1 Department of Medical Informatics, Biometry and Epidemiology, Ruhr University Bochum, Bochum, Germany; 2 Institute for Healthcare Management and Research, University Duisburg-Essen, Essen, Germany; 3 Alcedis GmbH, Gießen, Germany; 4 Centre for Sleep- and Telemedicine, Ruhrland-Clinic, University Medicine Essen, University Duisburg-Essen, Essen, Germany

**Keywords:** Mild to moderate position-dependent obstructive sleep apnoea, Sleep position therapy, Positive airway pressure therapy, Daytime sleepiness

## Abstract

**Background:**

First-line therapy for patients with clinically relevant obstructive sleep apnoea (OSA) is positive airway pressure therapy (PAP). At least one half of patients with mild to moderate OSA (apnoea-hypopnoea-index (AHI) 5-30/h) have positional OSA (POSA), where apnoea occurs mostly in supine sleep. These patients might benefit from sleep-position therapy (SPT) which should reduce sleeping time spent in a supine position. Until now, it is unclear whether SPT is non-inferior to PAP therapy for symptom relief in these patients.

**Methods:**

This is a multicentre, non-inferiority, open-label randomised, cross-over clinical trial. Patients with mild to moderate POSA and daytime sleepiness (according to Epworth Sleepiness Scale (ESS) > 10 points) will be randomised with a 1:1 allocation ratio, stratified by centre and AHI, to start with either PAP therapy or SPT and treated for 12 weeks. After a wash-out period of two weeks, they will switch to the other therapy for 12 weeks. The primary outcome is daytime sleepiness measured by the ESS at the end of each treatment phase. Analysis will be done in the intention-to-treat population using a linear mixed-effects model containing the intervention, the phase, the interaction between therapy and phase (including the carry-over effect) and the baseline measurement of the ESS and AHI as fixed effect, and centre and patient as random effects. A one-sided test at significant level of 2.5% will be used to test the non-inferiority of SPT with a non-inferiority margin of 1.35. Based on a sample size calculation with a one-sided one-sample t-test at significant level of 2.5%, assuming a standard deviation of 4, a total of 418 patients should be included to reach 80% power when SPT is only slightly inferior to PAP therapy (difference 0.8 resulting in a delta of 0.55 (1.35-0.8)). Assuming a 5% drop-out rate, 220 patients per sequence should be included. Possible futility stopping is planned at an interim analysis after 300 patients.

**Discussion:**

The recruitment of patients with mild to moderate POSA is feasible with the planned centres. Both certified interventions (PAP and SPT) are covered by the statutory health insurance companies as part of the trial guideline.

**Trial registration:**

DRKS00033048 registered 17. June 2024, http://www.drks.de.

## Administrative information

**Table:** 

Title {1}	Non-inferiority of sleep position therapy compared to positive airway pressure therapy with regard to daytime sleepiness in patients with mild to moderate position-dependent obstructive sleep apnoea (POSA): study protocol for a multicentre randomised cross-over trial
Trial registration {2a and 2b}	DRKS00033048
Protocol version {3}	24-Mar-2025, V3.0
Funding {4}	The trial has been commissioned by the German Federal Joint Committee (G-BA) to generate sufficient evidence for or against the reimbursement of sleep position therapy by the statutory health insurance funds in Germany. The Federal Joint Committee is the highest body of self-administration in the German healthcare system and determines which medical services those with statutory health insurance receive
Author details {5a}	Nina Timmesfeld^1^ Anja Neumann^2^ Frederik Valbert^2^ Jürgen Wasem^2^ Alexandra Spillner^3^ Christoph Schöbel^4^ Affiliations: 1 – Department of Medical Informatics, Biometry and Epidemiology, Ruhr University Bochum 2 – Institute for Healthcare Management and Research, University Duisburg-Essen 3 – Alcedis GmbH, Gießen 4 – Centre for Sleep- and Telemedicine, Ruhrland-Clinic, University Medicine Essen, University Duisburg-Essen
Name and contact information for the trial sponsor {5b}	**Sponsor:** University Duisburg-Essen, Universitätsstr. 2, Essen, Germany **Sponsor representative:** Prof. Wasem, Institute for Healthcare Management and Research, University Duisburg-Essen, Thea-Leymann-Str. 9, Essen, Germany, juergen.wasem@medman-uni-due.de, + 49 201 183–4072
Role of sponsor {5c}	The trial has been commissioned by the German Federal Joint Committee (G-BA) to generate sufficient evidence for or against the reimbursement of sleep position therapy by the statutory health insurance funds in Germany. The G-BA has developed a draft study design (see “Erprobungsrichtlinie”). The study team led by the University of Duisburg-Essen, who acted as sponsor, applied for the call for bids and was awarded the contract. The sponsor and the study team are responsible for the conduct of the study, in particular the selection of the study centres, data collection, management of the study, statistical analysis, interpretation of the data and writing the report. In particular, the study team finalised the study protocol with all details. The final German-language study protocol was approved by the G-BA. The study team must regularly report the status of the trial to the G-BA. The final study report has to be approved by the G-BA

## Introduction

### Background and rationale {6a}

Obstructive sleep apnoea (OSA) is the most common organic sleep disorder. During sleep, the relaxation of the muscles leads to a narrowing or collapse of the upper airways. In addition to the occurrence of snoring, this is accompanied by restricted or interrupted ventilation of the lungs. These events are called hypopnoea and apnoea. The associated oxygen drops in the blood lead to short arousals and thus to an interruption of sleep. Even if the arousals are often not remembered the next day, around 1/3 of OSA patients report relevant daytime sleepiness. Subjective daytime sleepiness can be determined using internationally validated questionnaires such as the Epworth Sleepiness Scale (ESS) [1]. In the medium to long term, the stress reactions caused by sleep-related breathing disorder also led to an increased risk of cardiovascular diseases, depression, and dementia [2]. After a specific medical history, patients with suspected OSA get an overnight portable breathing monitoring system (polygraphy = PG) at their homes in accordance with the German guideline on methods of contract medical care issued by the German Federal Joint Committee (G-BA) [3]. If there is a hint of a sleep-related breathing disorder, but a final diagnosis cannot be proven based on portable monitoring, the diagnosis is confirmed by a fully attended polysomnography (PSG) in a sleep laboratory.

The severity of OSA is defined by the apnoea-hypopnoea index (AHI)—i.e. the number of breathing disturbances per hour of sleep. Here, the normal value is an AHI < 5/h. A mild OSA is diagnosed with an AHI of 5– < 15/h, a moderate OSA with an AHI of 15– < 30/h, and a severe OSA with an AHI ≥ 30/h. Based on the AHI, current epidemiological studies show a high prevalence of OSA. In Germany, for example, around 14 million people are estimated to show an AHI ≥ 15/h and even around 26 million an AHI ≥ 5/h [4]. According to the S3 guideline of the German Society for Sleep Research and Sleep Medicine (DGSM), treatment is indicated for an AHI ≥ 15/h regardless of the associated symptoms or for mild OSA (AHI 5– < 15/h) with associated symptoms or an increased cardiovascular risk due to existing comorbidities [5].

The first-line therapy is positive airway pressure (PAP) therapy at night. During sleep, positive air pressure is delivered to the upper airways via a nasal or oronasal mask to keep them open. This effectively prevents obstructive sleep-related breathing disorders. This not only has a positive effect on daytime sleepiness, but also on the increased long-term risk of subsequent diseases. In line with the guideline, PAP therapy is initiated during a fully attended PSG in a sleep laboratory. This involves finding the optimum pressure setting that can keep the airways open. In most cases, a pressure range is set; the therapy device then automatically adjusts the necessary therapy pressure to the patient's needs during the night (automatic PAP therapy = APAP).

At least one half of patients with mild to moderate OSA have positional OSA, where apnea occurs mostly in supine sleep. In relation to the overall AHI averaged over the night, which shows a mild to moderate degree of OSA, the index in supine position is at least twice as high compared to the AHI in non-supine position [6]. Studies have shown that preventing supine sleep by means of sleep position therapy (SPT) can lead to a significant reduction in the overall AHI [7]. For example, SPT can be carried out using a position sensor, which is attached to the sleeper with an abdominal belt and in case of supine position uses gentle vibrations to encourage a change of position. Initially, sleep can be interrupted by this, so that a familiarisation phase of up to 4 weeks is assumed. During this time, a conditioning effect occurs, which should lead to avoidance of the supine position at night. Studies confirm the effect of such a therapy in terms of reducing sleep-related breathing disorders, but the effects on endpoints such as daytime sleepiness are not conclusively compared to established PAP therapy [6].

### Objectives {7}

The POSA trial is intended to answer the open question, whether SPT is at least as good as PAP therapy to reduce daytime sleepiness after 12 weeks of treatment in patients with mild to moderate positional OSA complaining relevant sleepiness assessed by Epworth-Sleepiness-Score > 10 points.

### Trial design {8}

**Fig. 1:**
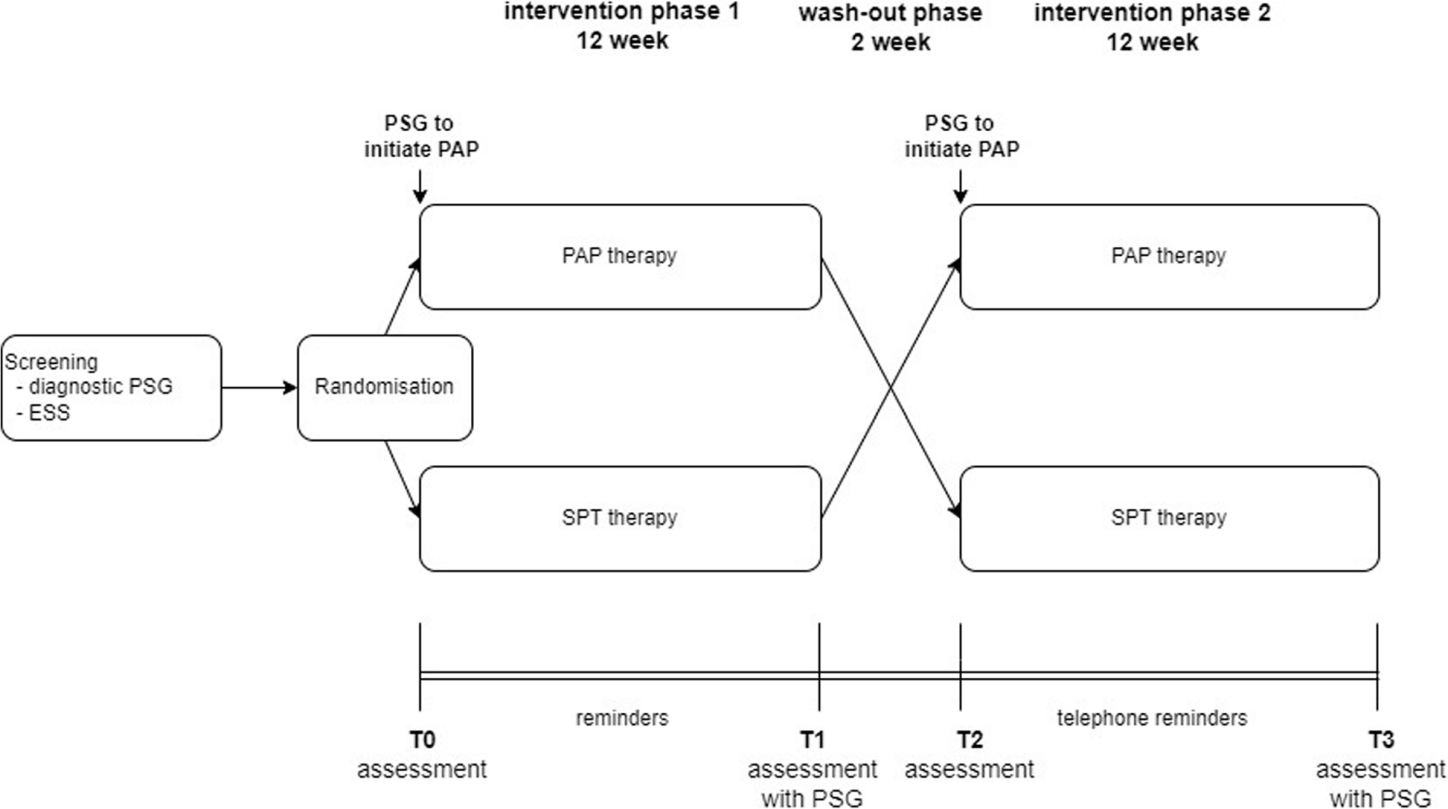
Study flow for the patients

This is a multicentre, individually randomised, controlled, non-inferiority trial with a cross-over design. Patients are randomised (1:1) to start with either PAP therapy or SPT and are treated for 12 weeks. After a subsequent wash-out period of 2 weeks, the study participants switch therapy groups and are treated with the other therapy for 12 weeks (see Fig. 1). Hence, there are two groups with different treatment sequences (1) SPT → PAP and (2) PAP → SPT. The length of the wash-out period was chosen based on recently published studies, in which the duration of this phase was only 0 or 1 week and yet there was no carry-over effect [6].

## Methods: participants, interventions and outcomes

### Study setting {9}

The study participants are recruited by specialists in sleep medicine or somnology in Germany. Patients who are referred to a sleep laboratory for diagnostic confirmation in case of suspected OSA according to clinical routine are screened for eligibility criteria. Based on the diagnostic polysomnography performed, the inclusion and exclusion criteria are checked by the investigators at the respective study centre. Furthermore, the medical devices for the interventions are given to the patients and are used at home.

### Eligibility criteria {10}

In order to participate in the trial, the following requirements should be met by the study centres. The study centres should be specialised in patients with sleep-related breathing disorders and must have access to a clinical sleep laboratory to perform fully attended polysomnography. In addition to qualified study personnel in accordance with GCP, a study physician with additional qualifications in sleep medicine or somnology who has experience in clinical sleep medicine studies should be on site. This ensures a high-quality study implementation in accordance with the protocol. It should also preferably be an outpatient sleep laboratory, as the patient population addressed is more likely to be expected and thus recruited in the outpatient care sector due to the inclusion and exclusion criteria.

Potential study participants are patients who are referred to a sleep laboratory for diagnostic confirmation of suspected OSA according to clinical routine and existing guidelines. Based on the diagnostic polysomnography performed, the following inclusion and exclusion criteria are checked by the investigators of the respective study centre. To ensure high quality, from each study centre up to five randomly selected PSG from the first inclusion nights should be verified by the Core Lab (Centre for Sleep and Telemedicine, University Medicine Essen). In the event of major deviations, the centre staff is trained by employees of the Core Lab.

Inclusion criteria:

1. Adult patients with age ≥ 18 years
2. Mild to moderate, supine-dependent obstructive sleep apnoea (total apnoea-hypopnoea index (AHI): 5– < 30/h
3. At least twice as high AHI in supine position compared to non-supine position
4. At least 30% of total sleep time in supine position
5. AHI in non-supine position: < 10/h)
6. Indication for nocturnal positive airway pressure therapy with relevant daytime sleepiness (Epworth Sleepiness Scale (ESS) > 10 points)

Exclusion criteria:

1. Previous or ongoing sleep apnoea therapy using equipment or surgery
2. Existing oxygen therapy
3. Active professional activity as a professional driver
4. Existing pregnancy
5. Taking sedative or vigilance-enhancing medication
6. Unstable medical condition which, in the opinion of the investigator, impairs participation in the study
7. Presence of other relevant sleep disorders affecting daytime sleepiness
8. Excessive alcohol consumption (> 3 drinks/day)
9. Drug use
10. Severe claustrophobia
11. Musculoskeletal problems that restrict sleeping in the lateral position
12. Inability to understand the study and patient information leaflet
13. Patients with an active cardiac device (pacemaker, AICD, CRT, etc.)
14. Use of other medical devices (e.g. implanted aggregates, neurostimulation procedures, etc.) that could be affected by the light vibration stimuli on the chest caused by the sleep position trainer
15. Health condition that requires sleeping in a supine position (e.g. due to shoulder or back surgery or osteoarthritis)
16. Upright sleeping position (e.g. by using more than two pillows)

### Who will take informed consent? {26a}

Based on the patient’s medical history and clinical findings (ESS score, ambulatory polygraphy findings), the eligibility of the patient for the study is determined by the study physician with additional qualifications in sleep medicine or somnology on site before the diagnostic PSG (first PSG in Fig. 1). If the patient is eligible for participation in the study based on these criteria, they will be informed about the study. As part of the information, the study is explained in detail regarding the background, the study design, the devices, the assessments, the handling of the participants’ data and the potential benefits for the participants. The investigators then obtain verbal and written consent from the study participants at the latest after the diagnostic PSG.

### Additional consent provisions for collection and use of participant data and biological specimens {26b}

Not applicable.

## Interventions

### Explanation for the choice of comparators {6b}

The first-line therapy for clinically relevant OSA is PAP therapy during sleep. PAP therapy is initiated in line with national guidelines during a fully attended polysomnography in a sleep lab. This involves finding the optimum pressure setting that can keep the airways open. In most cases, a pressure range is set; the therapy device then automatically adjusts the necessary therapy pressure (APAP) to the patient’s needs during the night. Once the optimal pressure range is set at the beginning of the 12-week period during sleep lab stay, it typically remains unchanged throughout the intervention phase. The APAP device continuously adjusts the delivered pressure within this predefined range in response to detected obstructive events during sleep (e.g. flow limitation, snoring, apnoeas, hypopnoeas). There is generally no routine adjustment of the pressure range by clinicians unless there are specific clinical concerns (such as insufficient treatment response or patient-reported side effects) prompting a reassessment. In our study, no protocolised or systematic re-titration or range adjustments were scheduled during the 12-week intervention.

### Intervention description {11a}

In SPT, a sensor is attached to the body via an abdominal belt: in case of sleeping in the supine position, SPT emits gentle vibrations that prompt the patient to change their sleeping position without disturbing their sleep. After the tenth night, when the vibration level has been gradually increased to reach the target stimulus intensity, this level remains constant during the remaining intervention period. The device continues to deliver vibrations at this intensity whenever the patient moves into the supine position. Similar to the APAP arm, no scheduled adjustments were made during the 12-week phase– especially as the stimulation settings cannot be changed with the SPTs used. The SPT is a certified medical product and begins by analysing sleep during the first two nights. From the third and up to the tenth night, light vibrations are then gradually increased to help the patient get used to the SPT device. The SPT mobile app allows the course of therapy to be tracked and accessed at any time.

### Criteria for discontinuing or modifying allocated interventions {11b}

Patients can withdraw their informed consent at any time without explanation. If the patient withdraws consent, no further data will be collected. The data already collected until that point will be stored and used for study purposes, if the participant does not request the deletion of their data. Data that has already been collected and included in analyses at the time of the request for data deletion remains unaffected by the deletion.

In case of proven failure of the above inclusion and exclusion criteria at baseline, the study participant may no longer participate in the study. This also applies if one of the exclusion criteria occurs during the course of the trial.

Study participants have the option to discontinue the intervention at any time without explanation. If a participant wishes to discontinue intervention, this is noted in the eCRF by the study staff, and the participant is asked for the reasons. In addition, the intervention can be discontinued by the attending physician in the event of intolerance of the intervention confirmed by the attending physician. Participants who discontinue intervention can continue to take part in the study and are then documented according to the protocol. They remain within the specified time schedule, i.e., in the event of a discontinuation of intervention during the 1 st intervention phase, the T1 and T2 visits take place at the specified times and, if desired, a cross-over to the respective comparative intervention in the 2nd intervention phase.

### Strategies to improve adherence to interventions {11c}

During the intervention phases, patients in both groups are contacted at one, two, four and eight weeks after intervention start to remind them to use the therapy regularly. To monitor adherence to the therapy, usage data of SPT or PAP therapy will be read out from the devices. The app of the respective device is used for this purpose, in which the data is saved regularly and can then be read out at the end of the delivery period.

### Relevant concomitant care permitted or prohibited during the trial {11d}

No other therapies for OSA are permitted.

### Provisions for post-trial care {30}

After the participants complete the trial, they will receive standard treatment according to the national guidelines, which is currently PAP therapy.

## Outcomes {12}

The primary endpoint of the study is the change in subjective daytime sleepiness, measured with the ESS [1] before and 12 weeks after the start of the respective intervention. The following parameters are to be analysed as secondary endpoints:

- Subjective sleep quality according to the Pittsburgh Sleep Quality Index (PSQI) [8]
- Objective sleep parameters derived from PSG in comparison to the initial diagnostic PSG-examination: Total AHI, proportion of sleep time in supine position, AHI in supine position, AHI in non-supine position, oxygen desaturation index (ODI), mean oxygen saturation (SaO2 mean), minimum oxygen saturation (SaO2 min), time with oxygen saturation < 90% (T90) (all assessed by pulse oximetry), Total sleep time (TST), sleep onset latency, total wake time after first falling asleep (WASO), sleep efficiency (SE), sleep times in sleep stages N1, N2, N3 and REM, heart rate variability, arousal index, respiratory disturbance index (RDI) [9]
- Health-related quality of life according to the Functional Outcomes of Sleep Questionnaire (FOSQ-10) [10]
- Quality of life according to EuroQol-5D-5L (EQ-5D-5L) [11]
- Adverse events (AEs) according to standardised severity classification and treatment discontinuations due to AEs
- Usage data of SPT or PAP therapy to evaluate therapy adherence and to analyse a usage-related therapy effect (here: daytime sleepiness), as sufficient and regular use of sleep apnoea therapy is a prerequisite for the therapy effect in relation to the baseline. For this purpose, a corresponding effectiveness analysis should be carried out (analysis of the actual utilisation time of the respective intervention in relation to the mean, specified total sleep time in the corresponding intervention phase). The utilisation data can be read out easily and securely via the SPT device application or via the PAP device’s internal data stored on a SD card or transmitted by telemonitoring.

### Participant timeline {13}

To screen patients, the ESS is assessed, and an initial polysomnography is performed. If a patient fulfils the inclusion criteria, informend consent is obtained, and all other assessments will be performed as given in Table 1. Further visits to the study centres are scheduled at the end of each therapy phase and at the beginning of the second therapy phase for the assessment of the endpoints (Table 1).

**Table 1:**
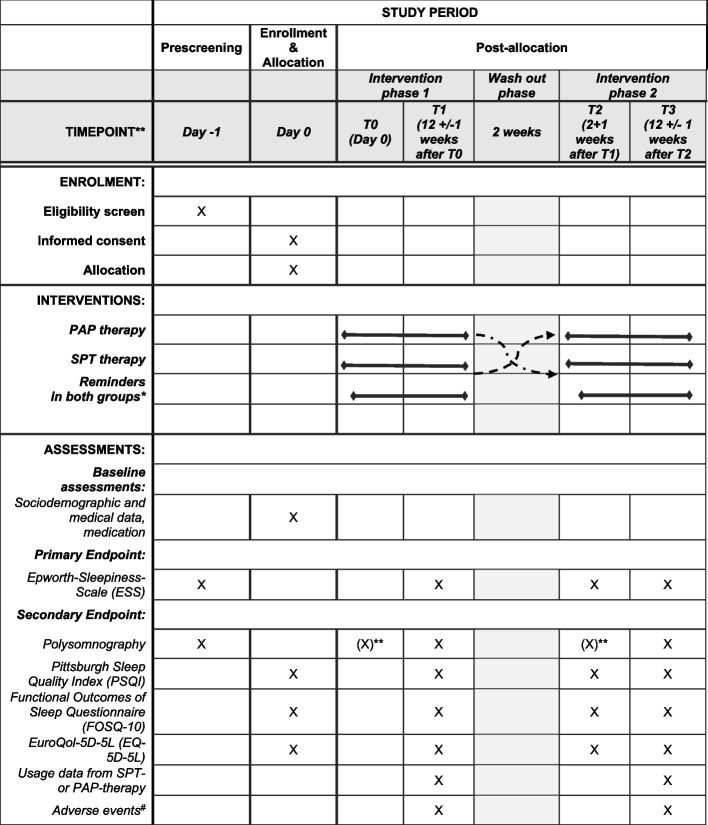
Schedule and timing of planned assessments

*At week 1, 2, 4, and 8 after T0 and at week 1, 2, 4, and 8 after T2; **only for controlled initiation of PAP therapy; ^#^additional assessments during reminder contacts

### Sample size {14}

The aim of the study is to show that SPT is not inferior to PAP therapy in terms of daytime sleepiness measured with the ESS. A difference of 1.35 between the two means is proposed as the non-inferiority threshold, i.e. the hypothesis pair:

$$H_0:\mu_{SPT}-\mu_{CPAP}\geq1.35\;vs.\;H_1:\mu_{SPT}-\mu_{CPAP}<1.35$$

should be tested with a one-sided test at significance level of 2.5%.

This means that under the alternative hypothesis, the ESS value measured under SPT may not be more than 1.35 points higher on average than the ESS value under PAP therapy. The choice of the value for the non-inferiority margin is justified as follows: The study by Crook et al. investigated the size of the clinically relevant difference for the ESS. Depending on the method used, the difference was between 1 and 4.21 [12]. In other non-inferiority studies, a difference of 1.5 or 2 points was often chosen as the non-inferiority threshold [13]. One meta-analysis showed a difference between PAP vs. no therapy of 2.4 points (95% CI, 1.9 to 2.8) [14]. However, a more recent meta-analysis showed that this difference depends, among other things, on the baseline ESS and AHI. For example, a difference of 1.34 (0.68 to 1.99) was found for a baseline ESS > 10 points and AHI 5 to < 15/h and 2.22 (2.13 to 2.30) for an AHI 15/h to < 30/h [15]. Therefore, the size of the non-inferiority margin of 1.35 provided in the documents of Federal Joint Committee seemed appropriate to ensure at least a difference compared to no therapy in the planned study population.

Hence, sample size calculation was done using a one-sided one-sample *t*-test at a significance level of 2.5% and to achieve a power of 80% to test the above hypotheses. It was assumed that the difference between SPT and PAP is 0.8 points. Sample size calculation for a δ of 0.55 points (1.35–0.8), assuming a standard deviation of 4, results in a required sample size of 418 patients in total, i.e. 209 per sequence. A drop-out rate of 5% is assumed according to the studies by Berry and Mok, i.e. revocation or completely missing data after baseline [6, 13]. Therefore, a total of 440 patients, i.e. 220 per sequence, will be recruited into the study. Further missing values do not affect the sample size, as they will be replaced according to the analysis strategy.

### Recruitment {15}

Recruitment was planned from Q4/2024 to Q2/2026 in approximately 15 German sleep centres. Before the start of the study, the centres were asked for an estimate of the number of patients who could be recruited during the study period. The total given from all study centres was approximately double the required sample size. As this trial is a cross-over study, it offers patients the opportunity to test both therapies, which is considered an advantage for recruitment.

Despite this, recruitment progress in the study sites is regularly monitored, and if a study site is significantly behind target, attempts are made to improve recruitment in consultation with the site. If this is not successful, this site will be closed and a new one opened.

## Assignment of interventions: allocation

### Sequence generation {16a}

Randomisation sequence (1:1 assignment) was generated using a computer-generated algorithm with randomly selected block sizes stratified by study centre and baseline AHI (mild vs. moderate) using the statistical software R, version 4.1.0 and the R package blockrand.

### Concealment mechanism {16b}

Randomisation will be done by the study physician via the electronic data capture system (EDCS, AlcedisTRIAL, Alcedis GmbH) after informed consent has been obtained from the study physician and the baseline data have been documented in the EDCS.

### Implementation {16c}

Allocation sequence will be generated by an independent statistician, who is not involved in enrolment and treatment of study participants.

## Assignment of interventions: blinding

### Who will be blinded {17a}

The study is an open labelled trial, so neither the patients nor the study physicians in the study centres are blinded to the therapy. The primary endpoint is self-reported by the patient, so it cannot be collected in a blinded manner. In addition, the statistical analysis will be done blinded; the evaluating statistician does not know the meaning of the group codes.

### Procedure for unblinding if needed {17b}

Since this is an open-labelled trial, no unblinding is necessary.

## Data collection and management

### Plans for assessment and collection of outcomes {18a}

Data from assessments and procedures will be collected following Table 1. All trial data will be collected through an electronic data capture (EDC) system. Project staff at the study centres will be trained in completing the electronic case report forms (eCRF) during initiation by the clinical research associates.

Patient reported outcomes (ESS, PSQI, FOSQ-10, EQ-5D-5L) are collected by paper and pencil and transferred to the EDC system by the study staff. Adherence data from the devices, for example nights and time used, are extracted from the device-specific app by the study staff and are then entered into the EDC system. There is no automatic import for this type of data to the EDC system due to the different manufacturers.

To ensure data quality of the primary and key secondary endpoints, on-site monitoring will be performed.

### Plans to promote participant retention and complete follow-up {18b}

To improve therapy adherence and participants’ retention, regular contacts via telephone calls or emails are planned. If it becomes clear during one of the contacts that the patient has dropped out of treatment, they are asked to participate in the outcome assessment, in particular of the primary endpoint, anyway. If the patient no longer wishes to come to the study centre, the primary endpoint can be collected by telephone. If drop-out of treatment was during the first therapy phase, the patient will be invited to receive the second therapy as planned.

### Data management {19}

For data capturing and data management of this clinical trial, a web-based validated software (AlcedisTRIAL) will be employed (eCRF). The development and setup is done by applying Good Automated Manufacturing Practice (GAMP) standards, fulfilling the FDA 21 CFR Part 11 and EU EudraLex V4 Annex 11 regulations. A set of Standard Operating Procedures (SOP) and guidelines are used during the study lifecycle project for supporting all study phases from specification, development, study start, deployment, and change management and up to study termination. To ensure high data quality, range checks will be made for numeric data at the time of data entry, and a warning will be displayed if the value does not meet the criteria. For categorical data, dropdown lists, radio buttons, and checkboxes will be preferred for data entry. All relevant fields, including all outcome measures, are marked as required fields so that data entry cannot be marked as complete before these data are captured; this will ensure collection of all outcome measures. The specifications of the checks applied to the data (both electronic checks and manual checks) are defined in the Data Validation Plan (DVP) including validation listings used for manual review. The DVP includes all definitions of electronic checks and the defined manual checks, which will be performed directly in the EDC system or can be based on listings. The data, as well as the medical review, are based on the defined manual checks of the DVP and are performed at regular intervals. Details of data management are defined in the data management plan.

The data processed by the Alcedis EDC systems is stored in a relational database. Specification and implementation of the database structure is based on the requirements of the particular study. The creation of the database is divided into several steps, which are specification, creation, field type verification, and structural verification. Any change in the database structure or its data is tracked. The database itself and its corresponding data at rest can be optionally encrypted. Access to the database is restricted.

Alcedis EDC systems are protected by mechanisms such as intrusion prevention and detection systems. Production data is separated from test and development data. The data processing takes place in data centres in Hessen/Germany. Encrypted backups are made daily and weekly. The daily backups are mirrored to a second data centre to prevent data loss in case of a local incident. Weekly backups are stored in a fireproof vault until they are delivered to a dedicated tape data archive. Data in transit, as when using the EDC system, is always encrypted.

### Confidentiality {27}

Pseudonymised personal data of participants will be stored in the EDC system (AlcedisTRIAL) in accordance with the German Federal Data Protection Act (BDSG). Data Access Groups (DAGs) will be used in the EDC system to restrict study staff access to data from their centre only. User rights will be restricted to the minimum necessary for individuals to perform their assigned tasks. Pseudonymisation is achieved through the use of unique centre numbers combined with consecutive patient numbers.

Re-identification can only be carried out in the study centre using the keylist. After the end of the trial, these keylists are destroyed as early as possible in accordance with the regulations. After the destruction, data will be fully anonymised.

### Plans for collection, laboratory evaluation and storage of biological specimens for genetic or molecular analysis in this trial/future use {33}

Not applicable, since no biological specimens have been collected.

## Statistical methods

The statistical analyses are carried out using the statistical software R (www.r-project.org), version 4.1.0 or higher. The analyses are blinded through the use of non-meaningful group codes, e.g. the groups are referred to as ‘Group A’ and ‘Group B’. For descriptive statistics, continuous variables are summarised using the arithmetic mean and standard deviation; in the case of a skewed distribution, the median and interquartile range are used. Categorical variables are summarised using totals and percentages.

Unless otherwise stated, for all confirmatory and exploratory analyses the estimate is given with the corresponding two-sided 95% confidence interval and the two-sided p-value. Two-sided *p*-values less than or equal to 5% or one-sided *p*-values less than or equal to 2.5% are considered statistically significant. The details of the planned statistical analyses will be specified later in the statistical analysis plan (SAP).

### Statistical methods for primary and secondary outcomes {20a}

#### Analysis sets

Three different analysis sets will be defined:

The *full analysis set* (FAS, based on the intention-to-treat (ITT) strategy) consists of all randomised patients according to the group allocation who fulfil the inclusion criteria and none of the exclusion criteria.

The *per-protocol set* (PPS) contains all patients from the FAS without serious protocol violations. The following protocol violations are categorised as serious:

- Discontinuation or intolerance regarding one of the two interventions
- Use of the respective therapy devices for less than an average of 4 h per night or on less than 50% of the nights analysed

This is not an exhaustive list. The full list of all serious protocol violations leading to exclusion from the PPS will be determined as part of the statistical analysis plan, taking into account the protocol violations that actually occurred during the conduct of the study. The final list of protocol violations will be finalised before closing the database and starting the statistical analysis.

The *Safety Analysis Set* contains all randomised patients.

#### Analysis for the primary and secondary endpoints

The aim of the study is to compare the use of the two therapies in terms of a treatment strategy. The difference in the ESS should therefore be determined without taking into account possible treatment discontinuations or compliance problems. For this reason, the analysis is conducted in a modified intention-to-treat population, which consists of all FAS patients with at least one value of the primary endpoint after baseline.

The *primary endpoint* is analysed using a linear mixed model. The model should contain fixed effects for the intervention, the phase, the interaction between therapy and phase (including the carry-over effect) and the baseline measurement of the ESS as well as the baseline AHI. Both the centre effect and the patient effect are modelled as random effects. The one-sided *p*-value corresponding to the hypothesis specified above, the estimated difference in ESS between the two treatments, and the corresponding one-sided confidence interval are reported. The one-sided *p*-value is calculated from the *p*-value of the above model either as p/2 or as 1-p/2 depending on the sign of the estimator. Details of the model, imputation of missing values, and sensitivity analysis are provided in the SAP.

The *secondary* quantitative endpoints are analysed using analogous models, with the difference that the baseline value of the endpoint under consideration is included in the model as a fixed effect instead of the baseline ESS. Analogous to the analysis of the primary endpoint, the p-value, the estimated difference, and the associated 95% two-sided confidence interval are also specified here. The analysis of dichotomous secondary endpoints is performed using mixed logistic regression models with the fixed effects for therapy, phase, and phase times therapy interaction as well as random effects for centre and patient. The analysis of count data, e.g. AHI, is performed with mixed Poisson models or, if necessary due to overdispersion, with negative binomial models with the fixed effects for treatment, phase, and phase times treatment interaction as well as random effects for centre and patient.

The secondary endpoints are analysed using two-sided tests at a significance level of 5%; all analyses of the secondary endpoints are exploratory.

### Interim analyses {21b}

A group-sequential approach is planned for the analysis of the primary endpoint, whereby only a futility stop is planned for the interim analysis based on the preliminary data. The studies by Berry and Mok both showed a mean difference in ESS of 2 points in the group with relevant daytime sleepiness (ESS at baseline > = or > 10) and thus in the clinically relevant inferior range [6]. For this reason, it should be possible to terminate the study prematurely if this clinically relevant inferiority was demonstrated. This interim analysis is planned after 300 evaluable patients. At this point, the power is 80% to demonstrate clinically relevant inferiority, i.e. ${H}_{1}:{\mu }_{SPT}-{\mu }_{CPAP}>1.35$, using a one-sided test at a significance level of 2.5%, assuming that the true effect is a 2-point difference in ESS. The corresponding 2.5% quantile for the standardised test statistic then serves as the futility limit.

The introduction of the futility limit reduces the significance level for the test of the primary hypothesis only insignificantly, so that adjustment is not necessary. This interim analysis therefore does not change the maximum number of cases, but it is possible to terminate the study prematurely if there is a clear effect in the SPT’s disadvantage.

### Methods for additional analyses (e.g. subgroup analyses) {20b}

The following subgroups should be considered: Mild OSA (AHI 5– < 15) vs. moderate OSA (AHI 15– < 30). The analyses in the subgroups are performed analogously to the analyses of the overall population.

### Methods in analysis to handle protocol non-adherence and any statistical methods to handle missing data {20c}

Due to the non-inferiority study, missing values for the primary endpoint resulting from treatment discontinuation and non-completion of the corresponding ESS questionnaire are replaced by the baseline value. In the case of missing values under both interventions, no imputation should be performed, as this is generally anti-conservative in non-inferiority studies. These are therefore omitted from the analysis, and the modified intention-to-treat population is analysed. In addition, sensitivity analyses with the most conservative possible replacement of the missing values under both conditions should be carried out.

The missing values are not replaced for the analysis of the secondary outcomes.

### Plans to give access to the full protocol, participant level-data and statistical code {31c}

The German study protocol will be supplied on request. The statistical analysis plan will be attached as a supplement to the publication of results. Any statistical code will be supplied on request. The data sets belong to the Federal Joint Committee (G-BA) and are available there on request.

## Oversight and monitoring

### Composition of the coordinating centre and trial steering committee {5d}

This trial has multiple collaboration partners, who are responsible for various tasks in the day-to-day study support: (1) the project management at Alcedis GmbH (AS) is available for all requests of the study centres; (2) data management at Alcedis GmbH is available for all questions relating to data capture and the eCRF; (3) lead principal investigator (CS) is available for all clinical questions; (4) biometrician (NT) is available for all methodological questions; (5) Sponsor representatives (AN,FV) are available for all organisational issues and reporting to the Federal Joint Committee (G-BA). The collaborators meet weekly to discuss the progress of the study, upcoming tasks, and open questions.

### Composition of the data monitoring committee, its role and reporting structure {21a}

Data and safety monitoring will be carried out by an external data safety monitoring board (DSMB) consisting of three persons (2 physicians, 1 biometrician). All board members will not otherwise be involved in the study. For the duration of the study, the DSMB will meet at least once after the above-mentioned interim analysis to enable a futility stop if necessary.

If the sponsor sees another reason why the decision of the DSMB should be obtained, an additional meeting may be initiated.

### Adverse event reporting and harms {22}

In the study, two established treatments with approved medical devices are used and compared within their respective intended application, whereby PAP therapy is already part of reimbursed standard care. The sleep position trainer is not yet part of reimbursed standard care but can be purchased (CE certified).

Serious adverse events are not expected. Possible adverse but transient effects may occur in connection with the sleeping position ‘forced’ by the SPT, inappropriately adjusted humidity during PAP therapy and/or too tightly fitting drawstrings of the sleep position trainer or the PAP face mask.

Study participants are closely monitored as part of the study to an extent that goes beyond standard care with PAP therapy. Adverse events will be recorded using the CTCAE (common terminology criteria of adverse events) severity classification and analysed in the study database. Serious incidents in accordance will be reported independently from the study in accordance with the Medical Device User Notification and Information Ordinance.

### Frequency and plans for auditing trial conduct {23}

Regularly scheduled plans for independent auditing the trial are not in place. However, a regular onsite monitoring is being scheduled in the treatment centres. The first monitoring visit should occur after the first randomised patient has finished the first study phase (approximately 3 months after randomisation). Approximately two visits per site are planned throughout the conduct of this clinical evaluation. After initiation, any site that enrolls at least 1 patient will get at least 1 monitoring visit. This visit may be combined with the Close-out visit.

### Plans for communicating important protocol amendments to relevant parties (e.g. trial participants, ethical committees) {25}

Regarding important protocol amendments, the ethics committees involved are informed about the amendment by the project management in coordination with the sponsor and G-BA. After approval, the sponsor will inform the principal investigators at study sites by the lead principal investigator and the project management (Alcedis GmbH). The principal investigators at study sites inform their research teams in detail about significant protocol modifications, and if necessary, notify the research participants.

### Dissemination plans {31a}

Study results will be disseminated via publication in peer-reviewed journals after study completion.

## Discussion

Recruitment of the planned number of patients with POSA of mild to moderate severity (according to AHI) and relevant daytime sleepiness should be feasible with the participating centres. As both interventions, PAP therapy and SPT, are certified medical devices, no serious adverse events are to be expected. As part of the cross-over study design, PAP therapy will be covered by statutory health insurance in line with routine clinical practice. SPT, which is currently not reimbursed in standard care, is also covered by statutory health insurers under the Federal Joint Committee's trial guideline. The statistical case number planning realistically allows the alternative therapy to be tested against the null hypothesis, i.e. to prove the non-inferiority of SPT compared to PAP therapy with regard to the primary endpoint “improvement in daytime sleepiness” in the addressed patient population.

## Trial status

Protocol version 2.0 was approved on May 22, 2024. The first centre was opened in September 2024, and the first patient is expected in October 2024. The enrolment is expected to be completed in December 2025. The trial in its entirety is expected to be completed six months after the last patient in, i.e. June 2026.

## Data Availability

Data collected for this study will be archived through Alcedis according to the current guidelines after finishing the analysis. Analysis of the data will be performed at the Department of Medical Informatics, Biometry and Epidemiology. When the study is completed, access to study data might be possible upon request to the G-BA.

## Abbreviations

AHI: Apnoea-hypopnoea index
APAP: Automatic PAP therapy
CTCAE: Common terminology criteria of adverse events
DGSM: German Society for Sleep Research and Sleep Medicine
DSMB: Data safety monitoring board
DVP: Data validation plan
eCRF: Electronic case report form
EDC: Electronic data capture
ESS: Epworth Sleepiness Scale
FAS: Full analysis set
G-BA: German Federal Joint Committee
ITT: Intention-to-treat
OSA: Obstructive sleep apnoea
PAP: Positive airway pressure
POSA: Positional obstructive sleep apnoea
PPS: Per-protocol set
PSG: Polysomnography
SAP: Statistical analysis plan
SPT: Sleep position therapy
